# Cost‐effectiveness of donepezil and memantine in moderate to severe Alzheimer's disease (the DOMINO‐AD trial)

**DOI:** 10.1002/gps.4583

**Published:** 2016-10-13

**Authors:** Martin Knapp, Derek King, Renée Romeo, Jessica Adams, Ashley Baldwin, Clive Ballard, Sube Banerjee, Robert Barber, Peter Bentham, Richard G Brown, Alistair Burns, Tom Dening, David Findlay, Clive Holmes, Tony Johnson, Robert Jones, Cornelius Katona, James Lindesay, Ajay Macharouthu, Ian McKeith, Rupert McShane, John T O'Brien, Patrick P J Phillips, Bart Sheehan, Robert Howard

**Affiliations:** ^1^ Personal Social Services Research Unit London School of Economics and Political Science London UK; ^2^ King's Health Economics Institute of Psychiatry, Psychology and Neuroscience, King's College London London UK; ^3^ Department of Biostatics Institute of Psychiatry, Psychology and Neuroscience, King's College London London UK; ^4^ Five Boroughs Partnership NHS Foundation Trust Warrington UK; ^5^ Wolfson Centre for Age Related Disorders King's College, London London UK; ^6^ Brighton and Sussex Medical School University of Sussex Brighton UK; ^7^ Campus for Ageing and Vitality Newcastle upon Tyne UK; ^8^ Birmingham and Solihull Mental Health NHS Foundation Trust Birmingham UK; ^9^ Department of Psychology Institute of Psychiatry, Psychology and Neuroscience, King's College London London UK; ^10^ Faculty of Medical and Human Sciences Institute of Brain, Behaviour and Mental Health, University of Manchester Manchester UK; ^11^ Division of Psychiatry and Applied Psychology University of Nottingham Nottingham UK; ^12^ Stratheden Hospital Cupar Scotland UK; ^13^ Faculty of Medicine University of Southampton Southampton UK; ^14^ Medical Research Council Clinical Trials Unit University College London London UK; ^15^ Division of Psychiatry University College London London UK; ^16^ Health Sciences University of Leicester Leicester UK; ^17^ Ayrshire and Arran NHS University Hospital Crosshouse Kilmarnock UK; ^18^ Institute for Ageing University of Newcastle Newcastle upon Tyne UK; ^19^ Oxford Health NHS Foundation Trust Warneford Hospital Oxford UK; ^20^ Department of Psychiatry University of Cambridge Cambridge UK; ^21^ Oxford University Hospitals NHS Foundation Trust Oxford UK; ^22^ Division of Psychiatry University College London London UK

**Keywords:** Alzheimer's disease, donepezil, memantine, cost‐effectiveness

## Abstract

**Objective:**

Most investigations of pharmacotherapy for treating Alzheimer's disease focus on patients with mild‐to‐moderate symptoms, with little evidence to guide clinical decisions when symptoms become severe. We examined whether continuing donepezil, or commencing memantine, is cost‐effective for community‐dwelling, moderate‐to‐severe Alzheimer's disease patients.

**Methods:**

Cost‐effectiveness analysis was based on a 52‐week, multicentre, double‐blind, placebo‐controlled, factorial clinical trial. A total of 295 community‐dwelling patients with moderate/severe Alzheimer's disease, already treated with donepezil, were randomised to: (i) continue donepezil; (ii) discontinue donepezil; (iii) discontinue donepezil and start memantine; or (iv) continue donepezil and start memantine.

**Results:**

Continuing donepezil for 52 weeks was more cost‐effective than discontinuation, considering cognition, activities of daily living and health‐related quality of life. Starting memantine was more cost‐effective than donepezil discontinuation. Donepezil–memantine combined is not more cost‐effective than donepezil alone.

**Conclusions:**

Robust evidence is now available to inform clinical decisions and commissioning strategies so as to improve patients' lives whilst making efficient use of available resources. Clinical guidelines for treating moderate/severe Alzheimer's disease, such as those issued by NICE in England and Wales, should be revisited. © 2016 The Authors. *International Journal of Geriatric Psychiatry* published by John Wiley & Sons Ltd.

## Introduction

Understanding the resource consequences of dementia treatments is particularly pertinent given projected increases in prevalence (Prince *et al.*, 2015) and associated expenditure (Comas‐Herrera *et al.*, 2007). Treatment decisions are increasingly informed by guidelines from bodies such as The National Institute for Health and Care Excellence (NICE), built on both clinical and cost‐effectiveness evidence. In 2009, NICE revised its guidance on acetylcholinesterase (AChE) inhibitor treatment of patients with moderate dementia, recommending that treatment should stop at the severe stage. Following new economic modelling, The National Institute for Clinical Excellence (2011) revised its guidance, allowing use of drugs within their licensed indications. A review of more recent evidence led to slightly updated guidance on medication treatment, with the three AChE inhibitors (donepezil, galantamine and rivastigmine) recommended as options for managing mild‐to‐moderate Alzheimer's disease (AD) and memantine recommended as an option for people with moderate AD who are intolerant of or have a contraindication to AChE inhibitors or with severe AD (National Institute for Health and Care Excellence 2016).

There remains little evidence to guide clinical decision‐making when patients reach moderate‐to‐severe AD. We therefore sought to examine the clinical and cost‐effectiveness consequences of continuing donepezil and commencing memantine (singly or in combination with donepezil). Clinical effectiveness findings have been published (Howard *et al.*, 2012); here, we examine the cost‐effectiveness consequences.

## Method

### Participants

Patients met standardised clinical criteria for probable/possible moderate or severe AD (McKhann *et al.*, 1984) and had been continuously prescribed donepezil for >3 months. Their prescribing clinician was considering change of medication, based on discussions with patient and carer, NICE guidance and clinical judgement. Patients had sMMSE score of 5–13 (Molloy and Standish, 1997), were community‐living and had a carer who was co‐resident or visited at least daily. Patients were recruited from 15 NHS English and Scottish centres between February 2008 and March 2010.

### Design

DOMINO‐AD was a multicentre, double‐blind, placebo‐controlled, factorial (2 × 2) clinical trial, with assessment of outcomes and costs over 52 weeks. It compared four treatments: (i) continue donepezil 10 mg per day with placebo memantine; (ii) discontinue donepezil (following 4 weeks donepezil 5 mg) with placebo memantine; (iii) discontinue donepezil and initiate memantine 20 mg per day; and (iv) continue donepezil 10 mg per day and initiate memantine 20 mg per day. Tablets were provided by the manufacturers. The study protocol was published before any data analysis (Jones *et al.*, 2009).

### Ethics

DOMINO‐AD was registered with the ISRCTN Registry (ISRCTN49545035). Ethical approval was received from Scotland ‘A’ Multicentre Research Ethics Committee.

An Independent Data Monitoring Committee reviewed efficacy and safety data every 6 months.

### Randomisation

A prepared unrestricted randomised list of assignments was used for the first 80 participants to ensure allocation concealment. Subsequent participants were randomly assigned to one of four treatment groups by the MRC CTU using randomised minimisation. Groups were stratified by centre, duration of donepezil treatment before entry (3–6 months; >6 months), baseline sMMSE score (5–9; 10–13) and age (<60; 60–74; >74 years). Patients, caregivers, clinicians, outcome assessors and investigators were blinded to assignment.

### Outcomes

Patients were assessed at baseline (pre‐randomization), at week 6 post‐randomisation to assess short‐term effects of donepezil withdrawal, and at weeks 18, 30 and 52.

Primary outcomes were:

- cognition: sMMSE (range 0–30, higher scores indicate better cognitive function) rated by treating clinicians;
- functioning in activities of daily living: Bristol Activities of Daily Living Scale (BADLS) (Bucks *et al.*, 1996) (range 0–60, higher scores indicate greater functional impairment) rated by carers.
- Secondary outcomes were:
- behavioural and psychological symptoms: Neuropsychiatric Inventory (NPI) (Cummings *et al.*, 1994) (range 0–144, higher scores indicate more symptoms) rated by treating clinicians;
- dementia‐specific health‐related quality of life: DEMQOL‐Proxy (Smith *et al.*, 2007) (range 31–134, higher scores indicate better quality of life) rated by carers;
- generic health‐related quality of life: EQ‐5D‐3L (EuroQol Group, 1990) rated by carers;
- health status of family or other unpaid carers: General Health Questionnaire (GHQ‐12) (Goldberg *et al.*, 1997) (range 0–12, higher scores indicate increased psychological morbidity).

The economic evaluation focused on three outcomes: sMMSE, BADLS, EQ‐5D‐3L. We applied societal weights to EQ‐5D‐3L (Dolan *et al.*, 1995) to calculate utility values. Quality‐adjusted life years (QALYs) were calculated by ‘area‐under‐the‐curve’ analysis, with linear interpolation between assessment points.

Before commencing data analysis, and based on the first 127 participants to complete DOMINO‐AD, the research team published values for minimum clinically important differences on sMMSE (1.4 points), BADLS (3.5 points) and NPI (8 points), based upon 0.4 standard deviations of changes from baseline (Howard *et al.*, 2011).

### Sample size

Original planned sample size was 800, adjusted to 430 based on standard deviations of outcomes from a blinded analysis of accrued data. Allowing for expected 20% missing visits, at two‐sided significance level of 5%, *n* = 430 would give 95% power to detect a 1.0 point sMMSE difference and 90% power to detect a 2.0 point BADLS difference between donepezil and placebo, or between memantine and placebo, at 52 weeks, and 96% power to detect a 1.5 point sMMSE difference, and 80% power to detect a 2.5 point BADLS difference between combination treatment and monotherapy at 52 weeks.

### Costs

Data on services and unpaid support were collected for each patient at:

- baseline (randomisation) for a retrospective period of 13 weeks;
- 6‐week post‐randomisation assessment retrospectively over 6 weeks;
- 30‐week assessment retrospectively over 24 weeks;
- 52‐week assessment retrospectively over 22 weeks.

Services and support data for patients were recorded on the Client Service Receipt Inventory (CSRI) (Beecham & Knapp, 2001) completed by family or professional carers, covering: inpatient stays, outpatient attendances, day hospital, social clubs, lunch clubs, day care, community‐based professional contacts (e.g. psychologists, psychiatrists, GPs, nurses, social workers, occupational therapists, home care) and all other services. Costs were assumed to be incurred by health and social care agencies even though some individuals make co‐payments. Data were also collected on volunteer support, befriending, telephone care‐line support and unpaid support by family and friends.

Unit costs reflecting long‐run marginal opportunity costs were drawn from available public sources, set at 2013/14 prices. Costs per unit of measurement for each service type (e.g. per contact with health professional) were mainly taken from Curtis (2014); NHS Reference Costs (Department of Health, 2014) were used for inpatient and outpatient attendances. Costs of unpaid care were estimated from information on volume and type of support, the opportunity cost of lost work (wage rate) for carers in paid employment and replacement cost for those not in paid employment based on cost of a home care worker (Curtis, 2014).

Medication costs were applied over the treatment titration and maintenance schedules. Donepezil came off patent in 2012 and memantine in 2014; we attached prices obtained from pharmacies at study sites for the generic versions (4p per 5‐mg tablet and 6p per 10‐mg tablet of donepezil; 4p per 10‐mg tablet and 7p per 20‐mg tablet for memantine). (At the time of the trial, both donepezil and memantine were under patent; donepezil prices were £2.27 per 5‐mg tablet and £3.18 per 10‐mg tablet, and memantine prices were £1.23 per 10‐mg tablet and £2.46 per 20‐mg tablet. However, we use *only* generic prices in our analyses)

### Cost‐effectiveness

Research questions were: (i) is donepezil continuation more cost‐effective than donepezil discontinuation over 52 weeks; (ii) is memantine, singly or in combination with donepezil, more cost‐effective than memantine placebo; and (iii) is the combination of donepezil and memantine more cost‐effective than donepezil alone.

Primary cost‐effectiveness analyses were conducted from a health and social care perspective. Cost subtotals were also calculated: trial medication costs; hospital costs (inpatient, outpatient, accident and emergency); and community‐based health, social and primary care. In sensitivity analyses, we adopted a societal perspective, adding unpaid care costs to health and social care service costs.

BADLS, SMMSE and QALYs were used, in turn, as measures of effectiveness in the cost‐effectiveness analyses.

### Statistical analysis

Analyses were conducted on participants receiving at least one dose of trial medication (including placebo), applying intention‐to‐treat principles as far as practicably possible, given missing data. Participants were analysed in groups to which they were allocated irrespective of treatment discontinuation or open‐label treatment. All non‐missing scores at every visit (irrespective of whether patients were still on trial medication or switched to open‐label treatment) were included; there was no imputation of missing scores. Full details of outcome analyses are given elsewhere (Howard *et al.*, 2012).

Cost data were analysed by regressing 52‐week health and social care costs (or societal costs in secondary analyses) on treatment allocation, centre, age at baseline, duration of donepezil treatment pre‐randomisation, baseline sMMSE and total health and social care costs (or societal costs) in the 13‐week pre‐randomisation period. To mitigate effects of data skewness, non‐parametric bootstrapping was used to estimate 95% confidence intervals (CIs) for mean costs. Where bias‐corrected 95% CIs of between‐group change scores excluded zero, they were judged significant at *p* = <0 · 05.

The cost‐effectiveness of one treatment over another was compared by calculating incremental cost‐effectiveness ratios (ICERs), defined as difference in mean costs divided by difference in mean effects. If one treatment had lower costs and better outcome than its comparator it was considered *dominant*. Difficulties arise when one treatment is both more effective and more costly than its comparator, leaving the decision‐maker to consider whether higher costs are justified by better outcomes. C*ost‐effectiveness acceptability curves* (CEAC) (van Hout *et al.*, 1994) were plotted for each cost‐outcome combination to show the likelihood of one treatment being seen as cost‐effective relative to another for a range of (implicit) values placed on incremental outcome improvements. Using the *net benefit approach*, monetary values of incremental effects and incremental costs were combined, and net benefit (NB) derived as:

$$NB\;=\;\lambda \;\times \;\left(\text{effec}t_{b}-\;\text{effec}t_{a}\right)\;–\;\left(cost_{b}–\;cost_{a}\right)\text{.}$$

λ is willingness‐to‐pay for a unit improvement in effectiveness (sMMSE, BADLS, QALYs), and a and b denote placebo and active treatment, respectively. A plausible range of λ values was explored for each outcome. This approach allows costs and outcomes to be considered on the same monetary scale, taking account of sampling uncertainty and adjusting for baseline covariates.

Analyses were undertaken using STATA (version 11) and SPSS 17.

### Sensitivity analysis

We explored consequences for cost‐effectiveness of adopting a societal rather than health and social care perspective.

## Results

### Sample

A total of 295 participants were recruited. Baseline characteristics were broadly comparable across treatments (Table 1). At baseline, full service use data and calculated costs were available for 291 (98.6%) participants (73 donepezil alone, 74 memantine alone, 72 donepezil–memantine combined, 72 placebo). By 52 weeks, health and social care cost data were available for 218 (73.9%) participants, and for *all* data collection points for 215 (72.9%). At 52 weeks, data on unpaid care were available for 186 (63.1%) participants and for *all* data collection points for 183 (62.0%). Thirty‐nine patients died over the trial period, one lost to follow‐up and 29 withdrew. Unless noted otherwise, analyses from a health and social care perspective are based on 215 individuals, and analyses from a societal perspective on 183.

**Table 1:** Baseline participant characteristics by treatment arm

		Donepezil alone	Placebo	Memantine alone	Donepezil plus memantine
*Total entered in trial*		*73*	*73*	*76*	*73*
Age in years/Mean		77.2	77.7	76.2	77.5
Gender; *n* (%)	Male	22 (30%)	26 (36%)	30 (39%)	24 (33%)
Previous duration of donepezil	3–6 months	3 (4%)	3 (4%)	4 (5%)	4 (5%)
	>6 months	70 (96%)	70 (96%)	72 (95%)	69 (95%)
Standardised Mini‐Mental State Examination (sMMSE)	Mean (sd)	9.0 (2.8)	9.1 (2.4)	9.2 (2.5)	9.1 (2.6)
Bristol Activities of Daily Living Scale (BADLS)	Mean (sd)	28.2 (9.0)	28.6 (8.9)	27.1 (9.0)	26.9 (9.8)
Neuropsychiatric Inventory (NPI)	Mean (sd)	22.3 (16.7)	22.9 (17.0)	23.1 (16.2)	20.3 (14.4)
DEMQOL‐Proxy	Mean (sd)	98.3 (13.5)	101.4 (11.7)	96.5 (15.3)	98.3 (13.5)
General Health Questionnaire (GHQ‐12)	Mean (sd)	2.3 (2.3)	2.8 (3.1)	3.1 (3.1)	1.8 (2.3)
EQ‐5D utility	Mean (sd)	0.57 (0.28)	0.55 (0.28)	0.59 (0.27)	0.55 (0.29)

### Outcomes

Effectiveness scores in Table 2 are not adjusted for baseline characteristics/centre; we *do* make adjustments for the cost‐effectiveness analyses below. Howard *et al.* (2012) detail the outcome analyses, where there was adjustment for the same variables except for pre‐randomisation costs. Additionally adjusting for this cost covariate very slightly changes some numerical values for differences between treatments but does not change conclusions about relative effectiveness.

**Table 2:** Clinical and quality of life measure scores (unadjusted for baseline characteristics) over time

	Donepezil alone	Placebo	Memantine alone	Donepezil plus memantine
	Mean (sd)	Mean (sd)	Mean (sd)	Mean (sd)
BADLS				
Week 6	29 (9)	32 (9)	28 (9)	28 (10)
Week 18	31 (11)	37 (9)	33 (9)	30 (10)
Week 30	33 (11)	38 (9)	34 (11)	31 (10)
Week 52	37 (11)	41 (9)	37 (10)	35 (9)
sMMSE				
Week 6	9 (4)	8 (4)	9 (4)	10 (4)
Week 18	8 (4)	5 (4)	8 (4)	9 (5)
Week 30	6 (4)	5 (4)	6 (4)	8 (5)
Week 52	5 (5)	3 (3)	5 (5)	6 (4)
Generic quality of life (EQ‐5D utility)				
Week 6	0.56 (0.28)	0.48 (0.28)	0.61 (0.26)	0.57 (0.28)
Week 18	0.52 (0.30)	0.40 (0.30)	0.52 (0.30)	0.56 (0.26)
Week 30	0.51 (0.32)	0.37 (0.29)	0.46 (0.29)	0.55 (0.26)
Week 52	0.48 (0.31)	0.26 (0.27)	0.42 (0.28)	0.49 (0.32)

Compared to patients randomised to donepezil discontinuation, those continuing on donepezil had higher sMMSE scores (mean 1.7 points; 95% CI 0.5 to 2.8) and lower BADLS scores (−2.9 points; 95% CI −5.3 to 0.5). In other words, adjusted comparisons suggest that both cognitive and functional impairment deteriorated less for patients remaining on donepezil compared to those who stopped. There was a greater QALY gain for the donepezil group compared to placebo (mean 0.11; 95% CI 0.02 to 0.20).

Compared to patients randomised to memantine placebo, those given memantine had higher sMMSE scores (mean 1.0 points; 95% CI −0.1 to 2.0) and slightly lower BADLS scores (−1.7 points; 95% CI −3.9 to 0.6), after adjustment for baseline covariates. There was no difference in QALY gain between these two groups (0.07; 95% CI −0.02 to 0.16).

The differences between treatment with donepezil alone and treatment with donepezil and memantine combined were not statistically significant after adjustment for covariates, in terms of sMMSE (mean 0.3 points; 95% CI −1.4 to 2.0), BADLS (−1.1 points; 95% CI −7.2 to 5.1) or QALY gain (0.02; 95% CI −0.19 to 0.22).

### Costs

Unadjusted health and social care costs were highest for people with placebo (£7964) and lowest for those with memantine alone (£4864); for people with donepezil–memantine combined, cost was £5892, and for donepezil alone £5418 (Table 3).

**Table 3:** Mean unadjusted costs (£, 2013/14 prices) of trial medication, hospital care, community‐based health and social care and primary care

	Donepezil alone		Placebo		Memantine alone		Donepezil plus memantine	
	Mean	(SD)	Mean	(SD)	Mean	(SD)	Mean	(SD)
Pre‐baseline (13 weeks)—*N*	*73*		*72*		*74*		*72*	
Hospital care	1516	(5289)	489	(2419)	327	(1037)	398	(1134)
Community‐based care	864	(2205)	925	(2852)	721	(2019)	647	(1932)
Total cost	2380	(5977)	1414	(3972)	1048	(2322)	1045	(2187)
Weeks 1–6—*N*	*72*		*71*		*73*		*68*	
Trial medication	3		1		4		5	
Hospital care	149	(643)	246	(1163)	254	(1012)	93	(394)
Community‐based care	248	(687)	242	(667)	149	(265)	221	(451)
Total cost	401	(939)	490	(1445)	414	(1122)	326	(695)
Weeks 7–30—*N*	*63*		*60*		*60*		*63*	
Trial medication	10		0		12		22	
Hospital care	1178	(4606)	1747	(4812)	764	(2512)	792	(1852)
Community‐based care	1396	(2607)	1709	(5877)	1121	(2525)	1556	(4222)
Total cost	2584	(5086)	3456	(7919)	1973	(3397)	2445	(4570)
Weeks 31–52—*N*	*54*		*55*		*51*		*58*	
Trial medication	9		0		11		20	
Hospital care	940	(2928)	597	(1729)	829	(2530)	758	(2323)
Community‐based care	2062	(3713)	3850	(17 929)	2124	(3854)	2069	(3547)
Total cost	3011	(4493)	4447	(17 944)	3033	(4580)	2915	(4365)
Weeks 1–52—*N*	*53*		*55*		*51*		*56*	
Total health and social care costs	5418	(7464)	7964	(23 707)	4864	(7416)	5892	(8607)

Unpaid care costs exceeded health and social care costs (Table 4). Societal costs were highest for people with memantine alone (£19 969), and lowest for those with donepezil–memantine combined (£16 058). Comparisons between these mean values should be tentative as they are not adjusted for baseline characteristics or centre.

**Table 4:** Mean unadjusted costs (£, 2013/14 prices) of health and social care and unpaid carer support, and total societal costs

	Donepezil alone		Placebo		Memantine alone		Donepezil plus memantine	
	Mean	(SD)	Mean	(SD)	Mean	(SD)	Mean	(SD)
Pre‐baseline (13 weeks)—*N*	*73*		*72*		*74*		*72*	
Health and social care	2380	(5977)	1414	(3972)	1048	(2322)	1045	(2187)
Unpaid care	4397	(7844)	2842	(5725)	4217	(6470)	5457	(7802)
Total societal cost	6777	(10 351)	4256	(6737)	5266	(6734)	6502	(7959)
Weeks 1–6—*N*	*72*		*71*		*73*		*68*	
Health and social care	401	(939)	490	(1445)	406	(1122)	319	(695)
Unpaid care	1257	(2144)	793	(1425)	1043	(1616)	1228	(2533)
Total societal cost	1658	(2319)	1283	(1888)	1449	(1846)	1547	(2613)
Weeks 7–30—*N*	*62*		*58*		*60*		*62*	
Health and social care	2178	(3967)	3222	(7733)	1898	(3397)	2311	(4583)
Unpaid care	4649	(8366)	2779	(5586)	4649	(6618)	4876	(5808)
Total societal cost	6828	(9254)	6001	(9167)	6547	(7634)	7186	(7922)
Weeks 31–52—*N*	*47*		*41*		*43*		*52*	
Health and social care	2870	(4202)	5603	(20 703)	3460	(4832)	2978	(4508)
Unpaid care	5385	(7341)	4659	(7312)	7111	(11 641)	3978	(5905)
Total societal cost	8256	(7725)	10 262	(22 558)	10 640	(12 556)	7024	(7844)
Weeks 1–52—*N*	*46*		*41*		*43*		*50*	
Health and social care	5530	(7592)	8531	(27 015)	5610	(7855)	6102	(8943)
Unpaid care	11 160	(15 035)	8884	(13 182)	14 359	(17 968)	9956	(11 815)
Total societal cost	16 690	(15 846)	17 415	(29 871)	19 969	(19 186)	16 058	(15 636)

We compared costs for patients allocated to each treatment after adjusting for centre, age at baseline, duration on donepezil pre‐randomisation, sMMSE at baseline and total costs prior to baseline (Table 5, top rows). There were no significant differences in health and social care costs or societal costs in any of the treatment comparisons.

**Table 5:** Mean adjusted cost differences, incremental costs and outcomes, and cost‐effectiveness ratios for each of the three treatment comparisons over weeks 1–52

	Mean difference* (95% CI)		
	Donepezil continuation versus donepezil discontinuation	Memantine versus memantine placebo	Donepezil and memantine combined versus donepezil alone
Mean adjusted cost differences (component and total) (£, 2013/14 prices)			
Medication costs	20	26	46
Hospital care costs	−63 (−1236 to 1110)	−594 (−1768 to 580)	−21 (−1761 to 1719)
Community‐based care costs	−196 (−3230 to 2839)	−1288 (−4465 to 1889)	93 (−1815 to 2001)
Unpaid care costs	−2037 (−4385 to 311)	−468 (−2467 to 1531)	−1875 (−4309 to 559)
Total health and social care costs	−389 (−3600 to 2822)	−1409 (−4912 to 2094)	599 (−2240 to 3438)
Total societal costs	−2669 (−7262 to 1923)	−1457 (−6330 to 3416)	−331 (−4641 to 3979)
Health and social care perspective: incremental costs and effects*, mean (95% CI)			
Costs	−389 (−3600 to 2822)	−1409 (−4912 to 2094)	599 (−2240 to 3438)
BADLS score	3.0 (0.7 to 5.2)	1.9 (−0.4 to 4.1)	0.8 (−3.5 to 5.2)
sMMSE score	1.7 (0.6 to 2.7)	0.9 (−0.1 to 1.9)	0.1 (−1.5 to 1.6)
QALY (EQ‐5D)	0.11 (0.03 to 0.19)	0.07 (−0.01 to 0.16)	0.03 (−0.10 to 0.16)
Health and social care perspective: incremental cost‐effectiveness ratios* (£, 2013/2014 prices)			
… for BADLS	Donepezil dominant	Memantine dominant	749
… for sMMSE	Donepezil dominant	Memantine dominant	5990
… for QALY	Donepezil dominant	Memantine dominant	19 967
Societal perspective: incremental costs and effects*, mean (95% CI)			
Costs	−2669 (−7262 to 1923)	−1457 (−6330 to 3416)	−331 (−4641 to 3979)
BADLS score	3.0 (0.7 to 5.3)	1.2 (−1.2 to 3.5)	1.1 (−2.5 to 4.7)
sMMSE score	1.5 (0.4 to 2.7)	0.6 (−0.6 to 1.7)	−0.3 (−1.8 to 1.1)
QALY (EQ‐5D)	0.09 (0.00 to 0.19)	0.02 (−0.08 to 0.12)	0.01 (−0.13 to 0.16)
Societal perspective: incremental cost‐effectiveness ratios* (£, 2013/2014 prices)			
… for BADLS	Donepezil dominant	Memantine dominant	301
… for sMMSE	Donepezil dominant	Memantine dominant	Donepezil dominant
… for QALY	Donepezil dominant	Memantine dominant	33 100

* Adjusted for centre, age, duration on donepezil, SMMSE score prior to randomisation and total costs at baseline.

** Higher scores indicate better outcomes on all measures

### Cost‐effectiveness analysis: health and social care perspective

Incremental cost‐effectiveness ratios (ICERs) for each outcome measure (BADLS, sMMSE, QALYs) were computed from a health and social care perspective (Table 5).

**Figure 1:**
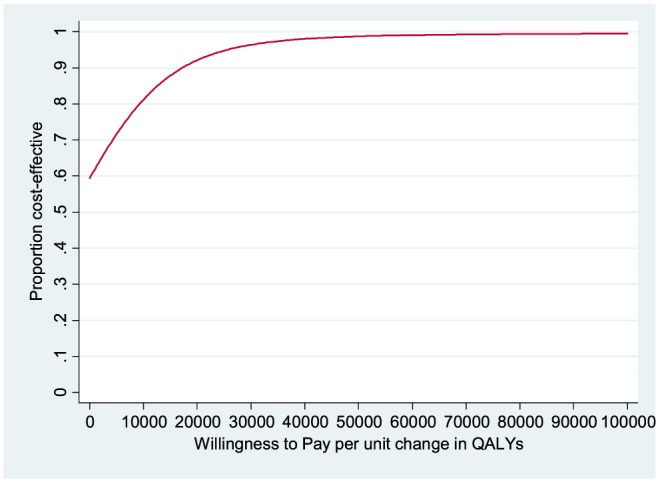
Cost‐effectiveness acceptability curve: donepezil continuation versus discontinuation; health and social care perspective, with effectiveness measured in QALYs. [Colour figure can be viewed at wileyonlinelibrary.com]

Patients continuing on donepezil had slightly lower but not significantly different costs than patients who discontinued donepezil. Given that donepezil continuation was associated with better outcomes than discontinuation, donepezil thus dominates discontinuation. CEACs allow us to summarise uncertainty in the estimates. The CEAC when outcome is measured by QALY gain (Figure 1) shows that the probability that donepezil continuation would be seen as more cost‐effective than discontinuation is 93% at the £20 000 threshold associated with NICE recommendations, and 96% at the £30 000 threshold (NICE, 2008).

**Figure 2:**
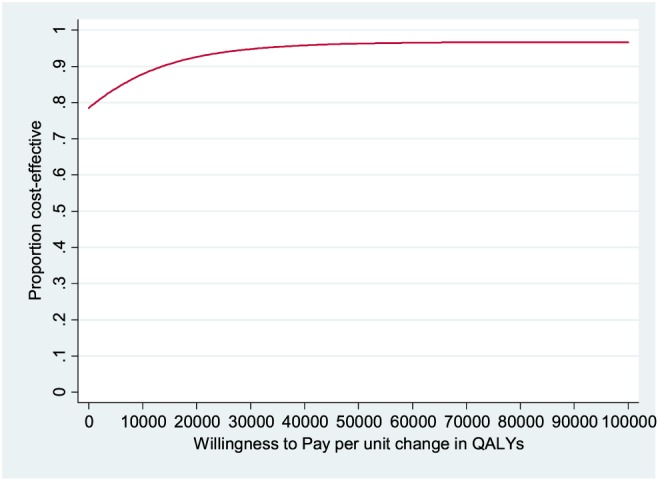
Cost‐effectiveness acceptability curve: memantine versus memantine placebo; health and social care perspective, with effectiveness measured in QALYs. [Colour figure can be viewed at wileyonlinelibrary.com]

For the second treatment comparison, patients treated with memantine (with or without donepezil continuation) had slightly lower but not statistically significantly different costs than patients treated with memantine placebo (with or without donepezil continuation), whilst clinical outcomes were close to being statistically significantly better (Table 5). Memantine dominates memantine placebo from a health and social care perspective, and the CEAC when outcome is measured by QALY gain suggests that the probability of memantine being more cost‐effective than memantine placebo is 92% at the £20 000 NICE threshold and 95% at the £30 000 threshold (Figure 2).

**Figure 3:**
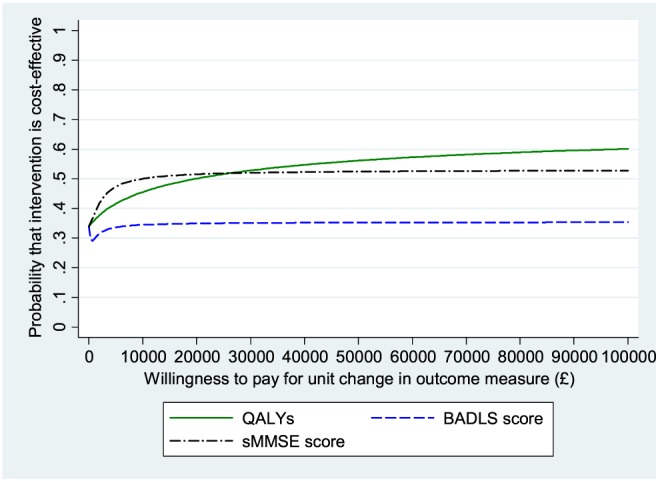
Cost‐effectiveness acceptability curves: donepezil and memantine versus donepezil only; health and social care perspective, with effectiveness measured in QALYs, BADLS and MMSE. [Colour figure can be viewed at wileyonlinelibrary.com]

The cost‐effectiveness analyses for the third comparison show that donepezil–memantine combined had slightly higher adjusted health and social care costs compared to donepezil alone, although the difference was not statistically significant. Cost per QALY gained was £19 967 (Table 5). An alternative way to summarise results for the other two outcome measures is to calculate average cost of achieving a minimum clinically important difference; for BADLS the annual cost of achieving a 3.5‐point difference is £2622, and for sMMSE the annual cost of achieving a 1.4‐point difference is £8386. The CEAC with QALY as outcome shows that the probability that donepezil–memantine combined would be seen as more cost‐effective than donepezil alone is only 50% at the £20 000 NICE threshold and 55% at the £30 000 threshold (Figure 3). For BADLS and sMMSE, the CEACs again suggest *low* probabilities that donepezil–memantine combined would be seen as more cost‐effective than donepezil alone, even at high willingness‐to‐pay values (Figure 3).

### Cost‐effectiveness analysis: societal perspective

We repeated the analyses from a societal perspective (Table 5). Outcome differences between treatments differ slightly between the rows in Table 5 because of the smaller sample with a societal perspective.

Donepezil continuation dominates donepezil discontinuation: better clinical outcomes and a reasonable, if not significant, societal cost advantage make donepezil continuation the more cost‐effective option.

Patients treated with memantine (with or without donepezil continuation) had slightly lower but not significantly different societal costs than patients treated with memantine placebo (with or without donepezil continuation), whilst clinical outcomes were not statistically significantly different. The CEACs (not shown) suggest reasonably high probabilities that memantine would be seen as more cost‐effective than memantine placebo when considering cognitive functioning (sMMSE, probabilities around 80%) or health‐related quality of life (QALY, probabilities around 73%) but low probabilities (below 20%) when considering BADLS.

In the third analysis, costs and outcomes were not observably different between donepezil alone and donepezil–memantine combined: cost and clinical outcome differences were tiny and not statistically significant. The probability that donepezil–memantine combined would be seen as more cost‐effective than donepezil alone is modest (e.g. below 60% for all willingness‐to‐pay values for QALYs).

## Discussion

Based on a double‐blind, placebo‐controlled trial of patients with moderate or severe AD already treated with donepezil, we found that continuation of donepezil treatment for a further 52 weeks was more cost‐effective than discontinuation. Donepezil's cost‐effectiveness was demonstrated regardless of whether outcomes were measured in terms of improvements in cognitive impairment, functional impairment or health‐related quality of life, and whether costs were measured just for the health and social care system or for society as a whole.

Starting memantine treatment was also more cost‐effective than donepezil discontinuation from a health and social care perspective by reference to all three outcome measures, and (though less strongly) was cost‐effective from a societal perspective when considering cognition and health‐related quality of life, but not when looking at functioning in activities of daily living.

In contrast, donepezil–memantine combined is not more cost‐effective than donepezil alone by reference to NICE thresholds for QALY gains, and the economic case also looks weak when considering the other two outcomes that we analysed (BADLS and MMSE).

### Previous studies

AChE inhibitors (such as donepezil) and memantine for the treatment of AD have been most frequently investigated for patients with mild‐to‐moderate symptoms. In moderate‐to‐severe AD (Feldman *et al.*, 2001; Tariot *et al.*, 2001) and severe AD (Fedldman *et al.*, 2005; Winblad *et al.*, 2006) AChE inhibitors are associated with modest improvements in cognition, function and clinical global impression. There is evidence that memantine is effective and cost‐saving in moderate and severe AD (Areosa *et al.*, 2005; Wimo *et al.*, 2003), but it is not clear whether memantine in combination with an AChE inhibitor confers additional clinical benefits (Tariot *et al.*, 2004; Porsteinsson *et al.*, 2008). However, there is little evidence to guide decisions regarding treatment continuation when symptoms become severe and patients are still living at home.

There is even less evidence on cost‐effectiveness. Few economic evaluations of Alzheimer's medications have been conducted within randomised controlled trials, although numerous studies have employed simulation models (Bond *et al.*, 2012; Knapp *et al.*, 2012). For donepezil, the only trial for patients with moderate‐to‐severe AD with an economic evaluation found no cost difference compared to placebo over 24 weeks from a health system perspective and modest savings (CDN$332) from a societal perspective (Fedlman *et al.*, 2001). For memantine, only modelling studies have been reported. For patients with mild‐to‐moderate dementia, AChE inhibitors enhance the effects of maintenance cognitive stimulation therapy and improve its cost‐effectiveness (D'Amico *et al.*, 2015).

Post‐hoc analyses of data from DOMINO‐AD showed that discontinuation of donepezil increased the risk of nursing home placement during the 52‐week trial period, although made no difference to this risk over the subsequent 4 years (Howard *et al.*, 2015). Nursing home admissions account for a relatively small part of the *overall* cost of AD, but delaying admission can substantially reduce service‐related costs for people with more severe dementia (Knapp *et al.*, 2016), even if it also risks increasing (prolonging) costs associated with unpaid care. We found that donepezil continuation was cost‐effective even when unpaid care costs were included.

The systematic review that informed the most recent NICE Guidance [UPDATE] on use of AD drugs identified very few trials with relevant data. The economic model found a probability of only 38% that memantine would be cost‐effective at a willingness‐to‐pay of £30 000 per QALY (Bond *et al.*, 2012), which is a lot lower than our finding here (94%). Our data therefore add to, and potentially change, the evidence base on the cost‐effectiveness of pharmacotherapy for people with moderate/severe AD by suggesting that both donepezil and memantine are more cost‐effective, when prescribed singly, than donepezil discontinuation.

### Limitations and strengths

DOMINO‐AD was unusual in evaluating medications for patients with more severe cognitive symptoms than has been common previously, specifically recruiting participants reaching the moderate‐to‐severe transition point. The study period of 52 weeks was unusually lengthy. The trial was publicly funded (MRC, Alzheimer's Society), and conducted independently. Both medications were covered by patent at the time of the trial, but are now both generic.

The trial failed to recruit its target number of participants (410). This did not affect the power to detect significant differences on the co‐primary outcomes (cognition and function) between donepezil and memantine and their respective placebos, but may have contributed to failure to demonstrate significant cognitive or functional benefits of donepezil–memantine combined over donepezil alone. The factorial design of the trial, however, allowed us to demonstrate the individual benefits of donepezil and memantine regardless of whether medications were taken alone or combined.

Estimating carer support costs is difficult: it is hard to measure time spent supporting someone with AD that is appropriately counted as ‘care’, and hard to attach an appropriate cost to that time. These common uncertainties in economic evaluation do not, of course, affect analyses from a health and social care perspective. We could not calculate carer costs for some patients, reducing sample size slightly for analyses from a societal perspective.

### Policy and practice implications

The independently conducted DOMINO‐AD trial offers new evidence on pharmacotherapy for AD patients who have progressed to the severe stage of their illness. The results have relevance for both clinical decision‐making (including decisions taken by health technology assessment bodies such as NICE) and for commissioning, given our findings on cost‐effectiveness. For example, NICE guidelines can only recommend the use of a medication within its licensed indication. AChE inhibitors are not currently licensed in England and Wales for the treatment of severe AD, but only for mild‐to‐moderate AD. Memantine is recommended for treatment of moderate or severe AD (National Institute for Health and Care Excellence 2016).

The trial suggests a strong case—not only on clinical grounds but also on economic grounds—for patients who have been successfully treated with donepzil at the mild‐to‐moderate stages but who have now progressed to more severe disease either continuing with donepezil or switching to memantine.

## Contributors

All authors contributed to study design, conduct of the trial and drafting of the paper. RH was Chief Investigator for the DOMINO‐AD trial and led the overall study. MK was lead investigator for the economic analysis, wrote the first draft of the paper and led all revisions. RR and DK carried out the analyses for this paper. All authors were involved in revision and approval of the manuscript for publication. The corresponding author (MK) had full access to all data in the study and had final responsibility for the decision to submit for publication. RJ was involved throughout the study and approved the previous version of this paper, but sadly died before the final version was completed.

## Conflict of interests

Dr Baldwin reports personal fees from Lundbeck, Otsuka, Pfizer, Novartis, Eli‐Lilly and Janssen‐Cilag, meeting expenses from Lundbeck, Otsuka, Pfizer and Eli‐Lilly, and paid participation in an advisory board for Lundbeck unrelated to the submitted work; Prof Ballard reports grants from Lundbeck and Acadia, and personal fees from Lundbeck, Acadia, Roche, Orion, GSK, Otsuka, Heptares and Lilly; Professor Banerjee reports research grants and personal fees from Abbvie, Eleusis, Daval International Ltd and Boehringer‐Indelheim, non‐financial support from Lilly; Dr Barber reports fees from Novartis outside the submitted work; Dr Bentham reports grants from the Medical Research Council during the conduct of the study, and personal fees from TauRx Therapeutics outside the submitted work; Professor Brown reports grants from the Medical Research Council and Alzheimer's Society; Dr Findlay reports grants from the Medical Research Council and Alzheimer's Society, personal fees from Eisai/Pfizer, Lundbeck, lecture fees and support to attend educational meetings outside the submitted work; Professor Holmes reports grants from the Medical Research Council and Alzheimer's Society, and a grant from Pfizer outside the submitted work; Professor Howard reports grants from the Medical Research Council and Alzheimer's Society UK, and non‐financial support from Pfizer/Eisai and Lundbeck during the conduct of the study; Dr Jones reports grants from the Medical Research Council and Alzheimer's Society and travelling expenses from Nottingham Healthcare Foundation Trust; Professor Katona reports honoraria and advisory board membership payments from Lundbeck outside the submitted work; Professor Knapp reports grants from the Medical Research Council and Alzheimer's Society, grant from Lundbeck outside the submitted work, personal fees from Lundbeck and Takeda outside the submitted work; Professor McKeith reports grants from the Medical Research Council and Alzheimer's Society, grants from Axavant Sciences and Nutricia outside the submitted work, personal fees from GE Healthcare outside the submitted work; Dr McShane reports grants from NIHR for the Cochrane Dementia and Cognitive Improvement Group outside the submitted work; Professor O'Brien reports personal fees from GE Healthcare, TauRx, Cytox outside the submitted work, grants and personal fees from Avid/Lilly outside the submitted work; Dr Phillips reports grants from the Medical Research Council during the conduct of the study; Drs Adams, Dening, Johnson, King, Lindesay, Macharouthu, Romeo, Sheehan have nothing to disclose.

## Funding sources

The study was funded by the UK Medical Research Council (G0600989) and UK Alzheimer's Society; part‐funded as independent research by the National Institute for Health Research (NIHR) Biomedical Research Centre and Dementia Unit at South London and Maudsley NHS Foundation Trust and King's College London (RBr and RH salary support) and the NIHR School for Social Care Research (MK salary support). Pfizer‐Eisai and Lundbeck donated medications and placebo. Neither funders nor pharmaceutical companies had any involvement in study design, management, conduct, data collection, analyses or interpretation of results.
